# Predicting Local Recurrence in T3 Laryngeal Squamous Cell Carcinoma: An Analysis of Tumor Volume and Cartilage Invasion Prior to Radiotherapy

**DOI:** 10.1002/cam4.71500

**Published:** 2025-12-26

**Authors:** Rasmus Blomkvist, Björn Palmgren, Eivind Gottlieb‐Vedi, Sara Jonmarker Jaraj, Antti Mäkitie, Linda Marklund, Lalle Hammarstedt‐Nordenvall

**Affiliations:** ^1^ Department of Clinical Science, Intervention and Technology, CLINTEC, Otorhinolaryngology Karolinska Institutet, and Karolinska University Hospital Stockholm Sweden; ^2^ Medical Unit, Head Neck, Lung and Skin Cancer Karolinska University Hospital Stockholm Sweden; ^3^ Upper Gastrointestinal Surgery, Department of Molecular Medicine and Surgery Karolinska Institutet, and Karolinska University Hospital Stockholm Sweden; ^4^ Department of Neuroradiology Karolinska University Hospital Stockholm Sweden; ^5^ Department of Otorhinolaryngology, Head and Neck Surgery University of Helsinki and Helsinki University Hospital Helsinki Finland; ^6^ Research Program in Systems Oncology, Faculty of Medicine University of Helsinki Helsinki Finland; ^7^ Department of Surgical Sciences, Section of Otolaryngology and Head and Neck Surgery Uppsala University Uppsala Sweden

**Keywords:** laryngeal cancer, laryngeal squamous cell carcinoma, recurrence, T3, volume

## Abstract

**Introduction:**

Patients with T3 laryngeal squamous cell carcinoma (LSCC) face a high risk of local recurrence after chemoradiotherapy (CRT) or radiotherapy (RT). This study examines whether tumor volume and inner cortex cartilage invasion (ICCI) predict local recurrence and survival.

**Materials and Methods:**

Eighty‐six patients with T3 LSCC, treated with RT or CRT at the Karolinska University Hospital between 2005 and 2021, were included. Cox regression assessed the effect of associations between tumor volume and ICCI on local failure and 5‐year mortality, adjusting for treatment modality, tumor subsite, and vocal cord mobility.

**Results:**

High tumor volume (> 5 mL) was associated with a threefold increase in local failure risk (HR 3.11, 95% CI 1.47–6.55). No significant associations were found between tumor volume and mortality, ICCI and local failure, or ICCI and mortality.

**Conclusion:**

The results demonstrate that a larger tumor volume correlates with a reduced treatment response for patients with T3 LSCC treated with RT or CRT.

## 1 Introduction

Despite advancements in the field of oncology, survival rates for laryngeal squamous cell carcinoma (LSCC) have not improved in recent decades, making it one of the few cancers globally with stagnant treatment outcomes [1]. The management is a balancing act between preserving the larynx and optimizing the treatment to reduce the risk of recurrence and death. Several organ‐preserving therapeutic strategies have been developed over time, such as radiotherapy (RT), chemoradiotherapy (CRT), partial laryngectomy, and transoral laser microsurgery, either as monotherapy or in combination. Although it has been suggested that patients with T4 LSCC treated with total laryngectomy (TL) have improved survival compared to T4 LSCC treated with organ‐preserving strategies [2, 3, 4], the optimal treatment for patients with T3 LSCC remains more controversial [5]. We recently reported better 5‐year overall survival and 5‐year recurrence‐free survival for patients with T4 LSCC at our center compared to patients with T3 LSCC (primarily treated with RT and CRT) [6], which indicates undertreatment in the latter group. Finding predictive markers for patients with T3 LSCC at high risk for being non‐responders to RT or CRT, and who would tolerate surgery as a supplemental primary treatment, could help to decrease the risk for local failure and increase survival rates.

Previous studies have indicated a reduced therapy response and survival for patients with large tumor volume treated with organ preservation strategies [7, 8]. Furthermore, the fact that T4 LSCC responds poorly to RT and that TL is preferred suggests that individuals with cartilage invasion may not be appropriate candidates for organ preservation [2]. This study hypothesized that tumors with a large volume, and/or tumors with inner cortex cartilage invasion (ICCI), have a greater risk of a reduced RT/CRT response and therefore a higher risk of local failure and decreased survival.

## 2 Materials and Methods

### 2.1 Study Design

This was a single‐center cohort study including all patients with T3 LSCC treated with curatively intended RT or CRT at the Karolinska University Hospital between 2005 and 2021. Different categories within the study parameters tumor volume (main) and ICCI (secondary) were compared regarding the outcomes local recurrence (main) and all‐cause 5‐year mortality (secondary).

The Swedish Head and Neck Cancer Register and the hospital archives were used to identify all patients with a diagnosis of T3 laryngeal cancer, according to the 7th edition of the TNM classification, managed at the Karolinska University Hospital during the time period May 1, 2005 until November 30, 2021, defined according to any of the following ICD‐10 codes: C32.0—malignant neoplasm of glottis, C32.1—malignant neoplasm of supraglottis, C32.2—malignant neoplasm of subglottis, and C32.8—malignant neoplasm of overlapping sites of larynx. Exclusion was made for patients with laryngeal carcinoma diagnosed prior to the study period, primary treatment with surgery, palliative or not completed curatively intended treatment, other histological subtypes than SCC, and no available computer tomography (CT) imaging (*n* = 2). Patient and tumor characteristics and treatment regimen details were extracted from the hospital records for each patient.

### 2.2 Treatment Protocol

Radiotherapy was administered using a fractionated regimen of two gray (Gy) per fraction, one fraction per day, 5 days per week. The median radiation dose in the cohort was 68 Gy (range 51–73 Gy). Chemoradiotherapy consisted of the same radiotherapy protocol, most commonly combined with concomitant cisplatin‐based chemotherapy, with or without prior induction therapy. Cetuximab was most frequently used in cases where cisplatin was contraindicated.

### 2.3 Study Parameters

The main study parameter was volume (milliliter; mL) of the primary tumor which was used to divide patients into two groups based on the median value, in order to preserve power (low: ≤ 5 mL [reference] and high: > 5 mL). To address the robustness of the selected tumor volume cut‐off, a sensitivity analysis was conducted using an alternative threshold of 3.5 mL (low: ≤ 3.5 mL [reference] and high: > 3.5 mL), which has been suggested as a possible clinically relevant cut‐off by previous research [9]. Tumor volume was manually segmented by a Head and Neck radiologist on contrast‐enhanced CT images using GE Health Care AW server. CT stacks of 2.5–3.0 mm were used and tumor area was manually traced for volume calculation.

The secondary study parameter was invasion of the inner cortex of the thyroid and/or adjacent cartilage. Invasion of the inner cortex was evaluated visually on contrast‐enhanced CT images by an experienced Head and Neck radiologist. The likelihood of ICCI was graded according to a four‐tier scale. Patients with ICCI (yes or likely) were compared to the reference group of patients without ICCI (no or not likely).

### 2.4 Outcomes

The main outcome was histologically verified local tumor recurrence during the follow‐up, referred to as local failure. The secondary outcome was all‐cause 5‐year mortality, that is, death from any cause within 5 years during the follow‐up.

### 2.5 Statistical Analysis

Risk of outcomes was visually depicted by Kaplan Meier curves and comparison between groups within each exposure was made by log‐rank test. Hazard ratios (HR) with 95% confidence intervals (95% CI) were produced for each study parameter and outcome using multivariable proportional Cox regression analysis. For the main outcome, local failure, time at risk was defined as date of diagnosis until date of verified local recurrence, death or end of follow‐up, whichever occurred first. For the secondary outcome, all‐cause 5‐year mortality, time at risk was defined as date of diagnosis until date of death or end of follow‐up (maximum 5 years), whichever occurred first. No patients were lost to follow‐up for either outcome. Analysis was made according to a crude model and an adjusted model including the three following possible confounding variables: primary treatment modality (radiotherapy or chemoradiotherapy), tumor site (glottic or supraglottic), and vocal cord mobility (normal, impaired, or fixed). Proportional hazards assumption was assessed by visual inspection of log–log curves. In additional explorative analyses to evaluate potential effect modification, an interaction term was included for smoking (current, former, and never) for the adjusted models for all study parameters and outcomes, with HRs derived for each smoking stratum. Complete case analyses were conducted, meaning only patients with no missing data for the included variables were included in the analyses. The statistical analyses were performed in the statistical software STATA IC 16.1 (StataCorp LCC).

### 2.6 Ethics Statement

The study was approved by the Swedish Ethical Review Authority (2019‐04829 and 2021‐06907‐02).

## 3 Results

Baseline characteristics of the included covariates and other selected variables are presented in Table 1. The cohort compromised a total number of 86 patients with glottic or supraglottic LSCC. No patients had subglottic LSCC. Median tumor volume was 5 mL (range 0.3–45.8). The group with high volume (> 5 mL) comprised a higher proportion of current smokers, low histological differentiation, supraglottic primary tumor site, N+ stage and fixed vocal cords compared to the group with low tumor volume. Thirty‐three patients had ICCI, 50 did not, and three had missing information and could thus not be included in the analyses. The group with ICCI had a higher proportion of patients with glottic tumor site, transglottic tumor growth, and fixed vocal cords.

**TABLE 1:** Baseline characteristics of patients with T3 laryngeal squamous cell carcinoma who completed radiotherapy or chemoradiotherapy with curative intent at the Karolinska University Hospital between 2005 and 2021.

	Volume		Inner cortex cartilage invasion (ICCI)	
	Number (%)			
	Low ≤ 5 mL	High > 5 mL	No	Yes
Total	44 (51)	42 (49)	50 (60)	33 (40)
Age, median (IQR)	69 (60–77)	64 (55–69)	66 (56–74)	68 (62–75)
Sex				
Female	5 (11)	11 (26)	10 (20)	4 (12)
Male	39 (89)	31 (74)	40 (80)	29 (88)
WHO				
0	21 (48)	23 (55)	25 (50)	16 (49)
1	16 (36)	10 (24)	14 (28)	12 (36)
2	7 (16)	6 (14)	8 (16)	5 (15)
3	0 (0)	3 (7)	3 (6)	0 (0)
Smoking status (at diagnosis)				
Current	25 (57)	37 (88)	39 (78)	21 (64)
Former	14 (32)	4 (10)	9 (18)	9 (27)
Never	5 (11)	1 (2)	2 (4)	3 (9)
Histological differentiation				
High	3 (7)	3 (7)	2 (4)	3 (9)
Moderate	24 (55)	13 (31)	21 (42)	16 (49)
Low	8 (18)	21 (50)	21 (42)	7 (21)
Missing	9 (20)	5 (12)	6 (12)	7 (21)
Tumor site				
Glottic	34 (77)	10 (24)	20 (40)	24 (73)
Supraglottic	10 (23)	32 (76)	30 (60)	9 (27)
N classification				
N0	39 (89)	25 (60)	35 (70)	27 (82)
N+	5 (11)	17 (40)	15 (30)	6 (18)
Subsites, tumor growth				
Glottic	5 (11)	0 (0)	2 (4)	3 (9)
Supraglotttic	7 (16)	20 (48)	19 (38)	5 (15)
Glottic + supraglottic	13 (30)	9 (21)	13 (26)	9 (27)
Glottic + subglottic	1 (2)	0 (0)	0 (0)	1 (3)
Transglottic	18 (41)	13 (31)	16 (32)	15 (46)
Vocal cord mobility				
Normal	20 (45)	10 (24)	20 (40)	8 (24)
Impaired	6 (14)	6 (14)	9 (18)	3 (9)
Fixed	18 (41)	26 (62)	21 (42)	22 (67)
Treatment				
Radiotherapy	25 (57)	20 (48)	25 (50)	20 (61)
Chemoradiotherapy	19 (43)	22 (52)	25 (50)	13 (39)
Local failure				
Yes	16 (36)	22 (52)	21 (42)	17 (52)
No	28 (64)	20 (48)	29 (58)	16 (48)

**FIGURE 1:**
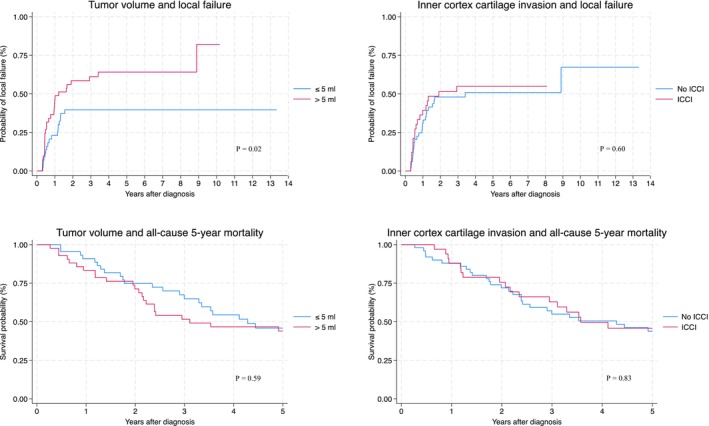
Kaplan–Meier curves illustrating the absolute risk of local failure and all‐cause 5‐year mortality by groups of tumor volume and inner cortex cartilage invasion (ICCI).

Median follow‐up time for the outcome local failure was 1.65 years (IQR 0.54–4.37) for ICCI and 1.79 years (IQR 0.54–4.44) for tumor volume. Median follow‐up time for the outcome all‐cause 5‐year mortality was 3.32 years (IQR 1.84–5.00) for ICCI and 3.44 years (IQR 1.78–5.00) for tumor volume. Absolute risk of local failure was increased for high compared to low tumor volume (*p* = 0.002), whereas no differences were observed for tumor volume and local failure, tumor volume and all‐cause 5‐year mortality, or ICCI and all‐cause 5‐year mortality (Figure 1).

A statistically significant two‐fold increased crude risk of local failure among patients with high volume T3 LSCC was observed compared to those with a low volume tumor (HR 2.01, 95% CI 1.09–3.68), which increased to three‐fold in the adjusted model (HR 3.11, 95% CI 1.47–6.55) (Table 2). Using the alternative cut‐off of 3.5 mL, high volume was associated with a somewhat lower but statistically significant increased risk of local failure (adjusted HR 2.39 [95% CI 1.15–4.96]) (Table 3).

**TABLE 2:** Hazard ratios with 95% confidence intervals and corresponding *p* values for the study parameters tumor volume and inner cortex cartilage invasion (ICCI) and the outcomes local failure and all‐cause 5‐year mortality.

	Volume		*p*	ICCI		*p*
	Low (≤ 5 mL)	High (> 5 mL)		No	Yes	
Local failure						
Crude	1.00 (ref)	2.01 (1.09–3.68)	0.02	1.00 (ref)	1.18 (0.64–2.17)	0.60
Adjusted^a^	1.00 (ref)	3.11 (1.47–6.55)	0.003	1.00 (ref)	1.13 (0.59–2.14)	0.72
Mortality						
Crude	1.00 (ref)	1.18 (0.66–2.11)	0.59	1.00 (ref)	0.94 (0.51–1.72)	0.83
Adjusted^a^	1.00 (ref)	1.80 (0.88–3.66)	0.11	1.00 (ref)	0.76 (0.40–1.43)	0.40

^a^ Adjusted for primary treatment modality (radiotherapy or chemoradiotherapy), tumor site (glottic or supraglottic), and vocal cord mobility (normal, impaired or fixed).

**TABLE 3:** Hazard ratios with 95% confidence intervals and corresponding *p* values for the study parameters tumor volume, with a cut‐off of 3.5 mL, and the outcomes local failure and all‐cause 5‐year mortality.

	Volume		*p*
	Low (≤ 3.5 mL)	High (> 3.5 mL)	
Local failure			
Crude	1.00 (ref)	1.88 (1.00–3.53)	0.05
Adjusted^a^	1.00 (ref)	2.39 (1.15–4.96)	0.02
Mortality			
Crude	1.00 (ref)	1.52 (0.82–2.82)	0.18
Adjusted^a^	1.00 (ref)	2.09 (1.00–4.38)	0.05

^a^ Adjusted for primary treatment modality (radiotherapy or chemoradiotherapy), tumor site (glottic or supraglottic), and vocal cord mobility (normal, impaired or fixed).

The HR for the adjusted all‐cause 5‐year mortality in the group with high tumor volume was 80% higher than in the group with low tumor volume (HR 1.80, 95% CI 0.88–3.66); however, it was not statistically significant (Table 2). On the contrary, using the cut‐off of 3.5 mL, high volume was associated with an increased risk of all‐cause 5‐year mortality (adjusted HR 2.09 [95% CI 1.00–4.38]) (Table 3). No statistically significant relationship was observed between ICCI and local failure (adjusted HR 1.10, 95% CI 0.58–2.10), or ICCI and all‐cause 5‐year mortality (adjusted HR 0.75, 95% CI 0.40–1.42) (Table 2).

A forest plot provided in Figure S1 provides the HRs with 95% CIs for all variables included in the multivariable Cox proportional hazards models (excluding tumor volume with the 3.5 mL cut‐off), together with the univariable analysis for nodal status (N+).

In the exploratory analyses in which effect modification by smoking was assessed, high tumor volume was associated with local failure in former and never smokers, but not in current smokers (Table S1). Moreover, high volume and ICCI were associated with both local failure and all‐cause 5‐year mortality only for never smokers. However, due to few patients in each group, especially among never smokers (low volume *n* = 5, high volume *n* = 1, no ICCI *n* = 2 and ICCI *n* = 3), results were non‐robust with high point estimates and wide confidence intervals.

## 4 Discussion

This single‐center cohort study including 86 patients with T3 LSCC treated with curatively intended RT or CRT showed a significantly increased risk of local failure for larger tumor volumes (> 5 mL). For patients with larger tumor volume, a tendency for increased all‐cause 5‐year mortality was observed; however, it was not statistically significant. Regarding ICCI, no association was observed with local failure or with all‐cause 5‐year mortality.

Results from previous studies regarding the optimal treatment of T3 LSCC are contradictory. While some large cohort studies have demonstrated that laryngeal preservation treatment with CRT offers equivalent survival rates compared to total laryngectomy (TL) [4, 10, 11], other studies have shown a superior survival for patients treated with surgery [12, 13]. In contrast, our previous study demonstrated poorer overall and recurrence‐free survival rates for glottic and supraglottic T3 LSCC (majority treated with RT/CRT), compared to T4 LSCC (majority treated with TL) [6]. Even though TL is not always the obvious solution, it was evident that patients with larger tumors treated primarily with surgery had a reduced risk of recurrence and a better OS [6]. Thus, there remains a need to identify patients who respond poorly to radiotherapy or who may not tolerate chemotherapy. Other treatment options for larger tumors already exist at some medical centers, such as different radiation doses [14] or customized transoral laser microsurgery [15, 16, 17]. However, the latter may depend on a high patient volume to maintain surgical competence, or treatment protocols may vary due to different traditions, making it inapplicable to many health care settings.

The results in this study were expected but are not applied in the current clinical cancer care guidelines. There are several studies indicating similar results. The cohort in our study was limited in size. This affects the precision and external validity of the results and moreover mean that only a few covariates could be adjusted for, possible leading to residual confounding. Moreover, the limited study size prevented us from performing subgroup analyses. Effect modification for smoking was assessed, but with non‐robust results. In addition, the cut‐off value based on the median value might not represent the most optimum cut‐off point but was used to maximize the power of the study. However, an association between tumor volume and locale failure, although weaker, was still observed when a lower tumor volume cut‐off value was used, indicating a possible dose–response relationship. A recently published study, also including T3 LSCC showed decreased disease‐free and overall survival per 1 mL increase in tumor volume [8]. A similar tendency regarding poorer overall survival was seen in the current study, however, it was not statistically significant. Another study showed an increased risk of loco‐regional failure if the tumor was larger than 3 mL [18]. Furthermore, an additional study showed a correlation between tumor volume above 2.5 mL, worse local control rate, and a decreased survival [7]. However, the oldest study included cases from 1966, after which diagnostics and treatment have evolved, which might be inapplicable in the healthcare setting of today.

ICCI or cartilage sclerosis has been evaluated in numerous earlier studies. Overall, CT seems to have a high accuracy in showing ICCI [19]. In some cases, though, it seems to underestimate inner cortex infiltration [19]. Magnetic resonance imaging (MRI), on the other hand, seems to have a higher sensitivity for ICCI but sometimes has a challenge of distinguishing between nontumor inflammatory changes and tumor infiltration, which results in an overestimation of suspected ICCI [20]. In our cohort, we only had CT images available which hence introduces the risk of misclassification of ICCI, which, however, is most likely to be non‐differential.

In concordance with previous studies, this single‐center study did not show any statistically significant correlation between invasion of the inner cortex of the cartilage and treatment response for patients with T3 LSCC [21]. A previous study with MRI showed no direct relationship with abnormal signal pattern in cartilage and prognosis, but when combined with a tumor volume > 5 mL it indicated a higher risk of recurrence [22]. In 2003, the tumor grading system was changed for larger LSCC and the definition of T4 was changed from tumors only engaging the cartilage to growing through the cartilage [23, 24]. The consequence of this is that some tumors that were previously assessed as T4 are currently defined as T3 and instead of surgery as the first treatment, these patients now receive RT or CRT. We had a theory that it was one of the reasons for the poor survival of T3 patients in our previous report [6]. However, no such trend was seen in our cohort.

This was a single‐center study, which reduces its external validity compared to multicenter studies. However, due to the Swedish health care system, all patients diagnosed and treated with LSCC in Stockholm during the study period were included, and none were lost to follow‐up. We did not adjust the data for changes in treatment regimens over time, which introduces a risk of bias by confounding. However, at the Karolinska University Hospital, the treatment protocols have been reasonably consistent during the time period of the study. The quality of CT images has improved over time and older CT images may have resulted in difficulties in estimating ICCI, which may have led to a misjudgment of cartilage involvement in earlier cases.

Even though tumor size appears to influence treatment response in this study, future larger and well‐conducted studies with adjustments for all relevant confounders on the topic are warranted. Tumor size alone may not be sufficient and should most likely be used in conjunction with other markers in order to identify patients who are not likely to respond well to RT.

## 5 Conclusion

Findings from this study indicate that a larger tumor volume is correlated with reduced treatment response in patients undergoing RT/CRT for T3 LSCC. Tumor volume has the potential to be used as a predictive marker in therapeutical decision‐making for the primary treatment approach in this patient population.

## Author Contributions

Rasmus Blomkvist: conceptualization, data curation, funding acquisition, investigation, methodology, project administration, visualization, validation, writing – original draft. Björn Palmgren: supervision, writing – review and editing. Eivind Gottlieb‐Vedi: formal analysis, methodology, writing – review and editing. Sara Jonmarker Jaraj: formal analysis, writing – review and editing. Antti Mäkitie: writing – review and editing. Linda Marklund: funding acquisition, methodology, supervision, writing – review and editing. Lalle Hammarstedt‐Nordenvall: conceptualization, methodology, writing – review and editing.

## Conflicts of Interest

Eivind Gottlieb‐Vedi reports consultancies for Sanofi, unrelated to the current study. Lalle Hammarstedt‐Nordenvall reports serving as a scientific review committee member for MSD, unrelated to the current study.

## Supporting information


**Figure S1:** Forest plot presenting hazard ratios with 95% confidence intervals for selected variables in relation to local failure and all‐cause 5‐year mortality. *Univariable analysis.
**Table S1:** Hazard ratios with 95% confidence intervals for the parameters tumor volume and inner cortex cartilage invasion (ICCI) and the outcomes local failure and all‐cause 5‐year mortality by effect modification of smoking.

## Data Availability

An exemption from the ethical committee was granted, allowing us to conduct the study without individual consent. However, the data are not publicly available as we do not have written consent from the participants.
