# Supplementary material for: Predicting Local Recurrence in T3 Laryngeal Squamous Cell Carcinoma: An Analysis of Tumor Volume and Cartilage Invasion Prior to Radiotherapy
Source: Cancer Med. 2025 Dec 26;15(1):e71500. doi: 10.1002/cam4.71500 (PMC12743200; doi:10.1002/cam4.71500)
Supplement: Supplementary file 1 — Figure S1: Forest plot presenting hazard ratios with 95% confidence intervals for selected variables in relation to local failure and all‐cause 5‐year mortality. *Univariable analysis. Table S1: Hazard ratios with 95% confidence intervals for the parameters tumor volume and inner cortex cartilage invasion (ICCI) and the outcomes local failure and all‐cause 5‐year mortality by effect modification of smoking. [file CAM4-15-e71500-s001.docx]

SUPPLEMENTARY MATERIALS

SUPPLEMENTARY FIGURE 1. Forest plot presenting hazard ratios with 95% confidence intervals for selected variables in relation to local failure and all-cause 5-year mortality. *Univariable analysis.

| SUPPLEMENTARY TABLE 1. Hazard ratios with 95% confidence intervals for the parameters tumor volume and inner cortex cartilage invasion (ICCI) and the outcomes local failure and all-cause 5-year mortality by effect modification of smoking. | | | | |
| --- | --- | --- | --- | --- |
|  | **Volume** | | **ICCI** | |
|  | Low (≤ 5 ml) | High (> 5 ml) | No | Yes |
| **Local failure** |  |  |  |  |
| Current smokers | 1.00 (ref) | 2.08 (0.92-4.70) | 1.00 (ref) | 0.97 (0.47-2.02) |
| Former smokers | 1.00 (ref) | 7.63 (1.70-34.26) | 1.00 (ref) | 2.02 (0.45-9.02) |
| Never smokers | 1.00 (ref) | 66.02 (3.36-1295.58) | 1.00 (ref) | 4.9e+08 (1.10e+08-2.18e+09) |
| **Mortality** |  |  |  |  |
| Current smokers | 1.00 (ref) | 1.44 (0.67-3.05) | 1.00 (ref) | 0.98 (0.49-1.92) |
| Former smokers | 1.00 (ref) | 2.19 (0.40-11.83) | 1.00 (ref) | 0.27 (0.05-1.42) |
| Never smokers | 1.00 (ref) | 629.67 (24.57-16139.05) | 1.00 (ref) | 1.01e+09(1.92e+08-5.31e+09) |
|  | | | | |
